# Lucinactant, a Synthetic Surfactant for the Treatment of Acute Respiratory Distress Syndrome Caused by COVID-19: Phase 1/2A Trial

**DOI:** 10.2196/77315

**Published:** 2026-07-31

**Authors:** Yuh-Chin Huang, Peter Morris, Robert Owens, Carlos Guardia, Steven Keller, Waldo Belloso, Lawrence Weinstein, Phillip Simmons, Steven Simonson

**Affiliations:** 1 Duke University Medical Center, 100 Duke Medicine Circle, Durham, NC, 27710, United States, 1 9196813394; 2 Department of Medicine, University of Alabama, Birmingham, AL, United States; 3 Department of Medicine, University of California, San Diego, La Jolla, CA, United States; 4 Windtree Therapeutics (United States), Warrington, PA, United States; 5 Department of Medicine, Johns Hopkins Hospital, Baltimore, MD, United States; 6 Department of Medicine, Hospital Italiano de Buenos Aires, Buenos Aires, Argentina

**Keywords:** surfactant, acute lung injury, lung compliance, oxygenation, COVID-19

## Abstract

**Background:**

One unique feature of COVID-19–associated acute respiratory distress syndrome (ARDS) is surfactant deficiency because SARS-CoV-2 specifically attacks alveolar type 2 cells.

**Objective:**

In this study, we evaluated the effects of a synthetic surfactant (lucinactant) therapy in patients with COVID-19–associated ARDS.

**Methods:**

This open-label multicenter phase 1/2A trial enrolled adult patients with COVID-19–associated ARDS who were within 7 days of mechanical ventilation initiation. Lucinactant (160 mL or approximately 80 mg of total phospholipids per kilogram of lean body weight) was delivered intratracheally. One retreatment was allowed at ≥6-hour intervals. The primary end points were oxygenation index and PO_2_-to–fraction of inspired oxygen ratio up to day 5 after dosing. Other end points included time to deliver lucinactant and respiratory system compliance (C_rs_). We monitored peridosing events and adverse events up to 30 days after dosing.

**Results:**

A total of 19 treated patients were enrolled (n=14, 73.7% receiving 1 dose; n=5, 26.3% receiving 2 doses). The mean age was 49 (SD 15) years. The mean time to administer lucinactant was 31 minutes. No significant changes were observed in oxygenation index (mean 10.4, SD 5.8 at baseline; 8.6, SD 2.7 at 12 hours; and 5.7, SD 2.4 on day 5; *P*=.12 via ANOVA) or PO_2_-to–fraction of inspired oxygen ratio (mean 193, SD 69 at baseline; mean 179, SD 57 at 12 hours; and mean 223, SD 105 on day 5; *P*=.44 via ANOVA). C_rs_ also did not change significantly (mean 40.7, SD 17.8 mL/H_2_O at baseline; mean 33.6, SD 8.5 mL/H_2_O at 12 hours; and mean 55.4, SD 18.5 mL/cmH_2_O on day 5; *P*=.12 via ANOVA). A total of 36.8% (n=7) of the participants died (6 from secondary infection and sepsis >13 days after dosing). In total, 15.8% (n=3) of the patients experienced transient peridosing desaturation or surfactant regurgitation.

**Conclusions:**

Our study showed that intratracheal instillation of 1 to 2 doses of lucinactant in COVID-19–associated ARDS was generally well tolerated. There were no significant changes in oxygenation parameters or C_rs_. The data suggest that, when future studies of lucinactant in patients with ARDS with severe surfactant dysfunction are conducted, an earlier and longer treatment protocol using different delivery methods that reach a larger alveolar surface area, such as aerosolization, may be needed to yield any beneficiary effects.

## Introduction

COVID-19, caused by SARS-CoV-2, was declared a pandemic by the World Health Organization on March 11, 2020. As of March 2024, there had been more than 770 million confirmed cases of COVID-19 and more than 7 million deaths reported to the World Health Organization [1]. COVID-19 manifestations range from mild (including some with no reported symptoms) to severe, including pneumonia and acute respiratory distress syndrome (ARDS), frequently leading to respiratory failure, requiring mechanical ventilation (MV), and death [2]. In 2020, ARDS-related deaths were 5 times the expected ARDS mortality predicted from previous years. The excess deaths were attributed to COVID-19 [3].

ARDS is a progressive pulmonary disorder characterized by lung inflammation and noncardiogenic pulmonary edema in association with hypoxemia, decreased lung compliance, and diffuse pulmonary infiltrates on the chest radiograph [4]. An influx of plasma proteins into the alveolar space results in surfactant dysfunction, worsening atelectasis of the lung and intrapulmonary shunt. ARDS may occur as a direct result of injury to the lung, such as pneumonia (both bacterial and viral), toxin inhalation, and aspiration of gastric content, or it may be associated with a wide variety of systemic processes such as sepsis, nonthoracic trauma, acute pancreatitis, multiple blood transfusions, fat embolism, or shock [5].

ARDS caused by COVID-19 not only has the pathophysiological features of surfactant inactivation by plasma proteins similar to non–COVID-19 ARDS but also has one unique feature: decreased surfactant production [6-9]. This is because SARS-CoV-2 preferentially attacks type 2 alveolar cells that contain the membrane angiotensin-converting enzyme 2 receptors [10]. Type 2 alveolar cells are the main source of surfactant in the lung, and if damaged, surfactant production is impaired. The secreted surfactant is further inactivated by autoantibodies [11]. Decreased production of surfactant along with inactivation of secreted surfactant results in severe surfactant dysfunction, a feature similar to neonatal respiratory distress syndrome (RDS) [6,7].

Surfactant supplementation has been used in previous pilot studies, showing some benefits in patients with non–COVID-19 ARDS [12,13]. However, subsequent large-scale multicenter trials [14-16] and a meta-analysis [17] failed to demonstrate a sustained benefit besides short-term improvement in blood oxygenation. The lack of efficacy has resulted in diminished clinical interest in the use of surfactant for ARDS in the past 2 decades. The emergence of COVID-19–associated ARDS reignited the interest in this therapeutic approach because of the direct effects of this infection on the surfactant system, resulting in primary surfactant deficiency similar to that of RDS [18,19]. One small trial used nebulized natural bovine surfactant and showed that the approach was feasible, but there was no significant improvement in oxygenation, secondary outcomes, or adverse events (AEs) [20]. Several other studies are still recruiting at the time of the study.

Lucinactant is a synthetic, non–animal-derived surfactant that contains phospholipids and neutral lipids, as well as a 21-residue synthetic peptide, sinapultide, which mimics the functions of surfactant-associated protein B [21]. Surfactant-associated protein B is made by alveolar type 2 cells and plays an essential role in spreading surfactant, lowering surface tension, stabilizing surfactant film, and promoting the recycling of surfactant. Lucinactant has one advantage over other surfactant preparations. Being a synthetic surfactant, it is less likely to trigger the immune reactions that can sometimes occur with animal-derived surfactant products. Lucinactant may possess anti-inflammatory effects [22]. Lucinactant is a US Food and Drug Administration–approved surfactant for the treatment of RDS in premature infants. It has been studied in several phase 2 and 3 clinical trials, showing superiority to some animal-derived surfactants in reducing the risk of RDS [23,24], bronchopulmonary dysplasia of the premature newborn [25], and acute lung injury in infants [26]. It has also been used in an ARDS treatment trial [13] where bronchoscopic delivery of surfaxin was associated with improvement in oxygenation.

As primary surfactant deficiency is a major pathobiological feature of COVID-19–associated ARDS, there is a strong rationale for replacement treatment with exogenous surfactant. Therefore, we designed a multicenter, international, single-arm treatment study to evaluate the feasibility, safety, and preliminary efficacy of lucinactant in adult patients with ARDS caused by COVID-19. We tested the hypothesis that a surfactant preparation with strong surfactant-associated protein B function can improve gas exchange and lung mechanics.

## Methods

This was a phase 1/2A multicenter, open-label, single-arm study conducted at 9 medical centers in 2 countries.

### Ethical Considerations

This study was approved by the institutional review boards of the University of Alabama Federalwide Assurance (UAB FWA 00005295); the University of California, San Diego (UCSD 210014); Duke University (Duke Pro00107651); and the Italian Hospital of Buenos Aires (Hospital Italiano #5191), as well as by each country’s respective regulatory authority. This study was conducted in accordance with current good clinical practice guidelines, the guiding principles of the Declaration of Helsinki, and applicable local laws and regulations. Study participants were unable to provide informed consent by themselves due to the severity of their illness; therefore, legal representatives were provided with institutional review board or ethics committee–approved informed consent forms for study participation. An independent data monitoring committee conducted preplanned safety reviews after every sixth participant was enrolled. The privacy and confidentiality of research subjects' data and identity were maintained.No compensation was provided to participants. The trial is registered at ClinicalTrials.gov (NCT04389671).

### Study Population

Participants were eligible for the study if they were aged 18 to 75 years, had ARDS from COVID-19 based on the Berlin criteria, were within 7 days of intubation for MV, and had a PO_2_-to–fraction of inspired oxygen (FiO_2_) ratio of ≤300. Patients with extremely severe lung disease (PO_2_-to-FiO_2_ ratio of <100 or oxygenation index [OI] of ≥25), severe renal impairment, or cardiac disease that adversely affected cardiovascular function, as well as patients with neuromuscular disease, active malignancy, and suspected concomitant bacterial or other viral lung infection, were excluded from the study. The complete inclusion and exclusion criteria are listed in Textbox 1.

Textbox 1. **Inclusion and exclusion criteria.**

**Inclusion criteria**

- Signed and dated informed consent form (in ink) by the patient or legally authorized representative
- Assay positive for SARS-CoV-2, preferably via polymerase chain reaction
- Endotracheal intubation and mechanical ventilation within 7 days of initial intubation
- In-dwelling arterial line
- PO_2_-to–fraction of inspired oxygen (FiO_2_) ratio of <300
- Mean blood pressure of ≥65 mm Hg with or without vasopressor support immediately before enrollment
- Bilateral infiltrates observed on frontal chest radiograph

**Exclusion criteria**

- Life expectancy of <48 hours or “do not resuscitate” orders
- Severe lung disease (home oxygen or forced expiratory volume in 1 second of <2 L) not likely to respond to therapy or profound hypoxemia (ie, oxygenation index of ≥25 or PO_2_-to-FiO_2_ ratio of <100)
- Severe renal impairment (creatinine clearance of <30 mL per minute)
- Having received within the previous 6 months or currently receiving immunosuppression therapy (azathioprine, cyclophosphamide, or methotrexate) or being a transplant recipient
- Clinically significant cardiac disease that adversely affected cardiopulmonary function: acute coronary syndromes or active ischemic heart disease (as assessed by the principal investigator using troponin and an electrocardiogram), cardiac ejection fraction of <40% (if known), need for multiple-dose vasopressors to support blood pressure (single-dose vasopressors, such as Levophed of ≤0.1 μg per kilogram per minute were allowed), cardiogenic pulmonary edema as the etiology of the current respiratory distress, or evidence of myocarditis or pericarditis
- Neuromuscular disease
- Neutropenia (absolute neutrophil count of <1000)
- Active malignancy that impacted treatment decisions or life expectancy related to this trial
- Suspected concomitant bacterial or other viral lung infection (bacterial infection defined as white blood cell count of >15,000 and positive blood, urine, or sputum culture results within 72 hours)

### Study Surfactant and Intervention

Lucinactant (Windtree Therapeutics, Inc) was provided as a lyophilized powder in a 30-mL vial and reconstituted using 10-mL sterile water for injection before administration.

Treatment was initiated within 6 hours of enrollment into the study. The study intervention consisted of direct, intratracheal delivery of lucinactant via the endotracheal tube (ETT) at a dose of 160 mL (approximately 80 mg of total phospholipids per kilogram of lean body weight). Before drug administration, the ventilator was set to an FiO_2_ of 1.0, and the use of sedation and short-acting paralytics was strongly recommended. The full dose was divided into 4 equal aliquots of 40 mL and administered through a catheter inserted in the ETT just above the carina. The first aliquot was administered with the participant in a supine position to the right mainstem bronchus. The participants were sequentially turned to the right decubitus position at 45° for 5 minutes to facilitate the distribution of the surfactant. Participants were closely monitored during lucinactant administration (blood pressure, heart rate, and peripheral oxygen saturation [SpO_2_]). If no peridosing events occurred, the second aliquot was administered to the left mainstem bronchus, and the participant was turned to the left at 45° for 5 minutes. The third and fourth aliquots were then administered to the right and the left mainstem bronchus, respectively, following the same procedures. If peridosing events were observed after 1 aliquot, the participants were ventilated until SpO_2_ recovered (>90%) before the next aliquot was administered.

Up to 3 retreatments of the same dose volume were allowed if the following criteria were met: participants remaining on MV with a PO_2_-to-FiO_2_ ratio under 300 no sooner than 6 hours after the previous dose and no evidence of pneumothorax. Retreatments were encouraged but not mandated and were administered at the discretion of the investigators. MV management and all other aspects of clinical care were left up to the study site clinicians.

### Clinical End Points

The primary efficacy end point was the change from baseline in OI (mean airway pressure × FiO_2_ × 100/PO_2_) and PO_2_-to-FiO_2_ ratio to study day 5. Other study assessments included change from baseline to study day 5 in SpO_2_, PO_2_, partial pressure of carbon dioxide in arterial blood (PaCO_2_), end-tidal carbon dioxide, oxygen saturation–to-FiO_2_ ratio, and respiratory system compliance (C_rs_). Ventilator-free days, days in the intensive care unit (ICU), days in the hospital, and organ failure–free days were also calculated. All clinical end points were collected at prespecified time points by the study coordinators. Because the participants were in strict isolation and may become clinically unstable, which affected on-time collection of data, we allowed the data to be collected within 2 hours of the prespecified time point.

Safety end points were assessed throughout the study and included all-cause mortality, peridosing events (desaturation, bradycardia, hypotension, and ETT reflux), assessment of vital signs, and the incidence of AEs. AEs were followed up on throughout the hospitalization period and, if ongoing, for an additional 30 days. Causal relationship to the study intervention was determined by the principal investigator at each site. An independent data monitoring committee met after every sixth participant had been enrolled and dosed.

### Statistics

This was an open-label, single-arm study with no control group. The objective of this safety pilot study was to determine whether lyophilized lucinactant administration was safe and tolerable and assess the impact on oxygenation and respiratory system compliance (C_rs_). As a result, no formal sample size calculation was performed. The original plan was to enroll up to 30 participants (out of approximately 90 screened).

All efficacy parameters were expressed as means and SDs. Descriptive statistics were calculated for parameters including changes from baseline to day 5 (OI, FiO_2_, PO_2_, SpO_2_, PaCO_2_, end-tidal carbon dioxide, PO_2_-to-FiO_2_ ratio, oxygen saturation–to-FiO_2_ ratio, plateau pressure, peak inspiratory pressure, positive end-expiratory pressure, ventilation index, and C_rs_). Descriptive statistics were calculated for ventilator-free days, days in the ICU, days in the hospital, organ failure–free days, and all-cause mortality. One-way ANOVA followed by the Dunnett subtest was used to compare OI, PO_2_-to-FiO_2_ ratio, and C_rs_ at 12 hours, 24 hours, and 5 days after treatment with values before treatment. The statistical analyses were performed using JMP Pro (version 17.2.0; JMP Statistical Discovery LLC).

## Results

The recruitment period of this study coincided with the Delta and Omicron COVID-19 variants. The study was terminated early because the number of patients with COVID-19–associated ARDS declined significantly since the Omicron variant wave began in November 2021. Between November 2020 and January 2022, a total of 23 patients were screened in 9 clinical sites. Of those 23 patients, 20 (87%) were enrolled in the study. Baseline characteristics are shown in Table 1. A total of 5% (1/20) of the enrolled participants did not receive the study treatment because of sudden deterioration before lucinactant administration and were excluded from outcome analysis. The remaining 95% (19/20) of the participants received at least one dose, and 25% (5/20) received repeated treatments. The mean time to administer the full dose via the ETT was 31 (range 10‐90) minutes.

**Table 1.** Baseline characteristics (N=20).

Characteristic and categories	Values
Gender, n (%)	
Male	16 (80)
Female	4 (20)
Race, n (%)	
White	12 (60)
African American	5 (25)
Others	3 (15)
Ethnicity, n (%)	
Hispanic	7 (35)
Non-Hispanic	13 (65)
Age (y)	
Median (IQR)	51 (36-58)
Mean (SD)	49 (15)
Age group (y), n (%)	
18-35	4 (20)
36-45	4 (20)
46-55	6 (30)
56-65	3 (15)
>65	3 (15)
Weight (kg), mean (SD)	117
Cardiovascular conditions, n (%)	
Any cardiovascular conditions	16 (80)
Hypertension	11 (55)
Respiratory conditions, n (%)	
Any respiratory conditions	7 (35)
Asthma	3 (15)
Metabolic conditions, n (%)	
Any metabolic conditions	19 (95)
Diabetes	7 (35)
Obesity	10 (50)
Hyperlipidemia	5 (25)
COVID-19–related medications, n (%)	
Any corticosteroids	17 (85)
Corticosteroids before lucinactant	16 (80)
Corticosteroids before and after lucinactant	5 (25)
Antivirals (remdesivir)	5 (25)

Table 2 summarizes the results of the primary and secondary end points. No significant changes were observed in OI (mean 10.4, SD 5.8 at baseline; mean 8.6, SD 2.7 at 12 hours; and mean 5.7, SD 2.4 on day 5; *P*=.12 via ANOVA) or PO_2_-to-FiO_2_ ratio (mean 193, SD 69 at baseline; mean 179, SD 57 at 12 hours; and mean 223, SD 105 on day 5; *P*=.44 via ANOVA). PO_2_ and PaCO_2_ remained stable throughout the 5-day period after dosing. C_rs_ was 40.7 (SD 17.8) mL/H_2_O at baseline, 33.6 (SD 8.5) mL/H_2_O at 12 hours, and 55.4 (SD 18.5) mL/H_2_O on day 5 (*P*=.12 via ANOVA). Other gas exchange end points, including PaO_2_-to-FiO_2_ ratio and PO_2_, showed no changes throughout the 5-day period after dosing (*P*=.14 and *P*=.43, respectively). There were also no changes in clinical outcome measures, including days on MV, days in the hospital, days in the ICU, and MV-free days.

Initial treatment was interrupted in 10.5% (2/19) of the participants and discontinued in 5.3% (1/19) of the participants due to peridosing events (desaturation and surfactant regurgitation). Study participants experienced a total of 54 AEs. Most of the AEs (51/54, 94%) were reported as nonrelated or unlikely to be related to the study intervention. A total of 6% (3/54) of the events (desaturation, retching, and worsened arterial blood gas) were reported as possibly related. In total, 32% (17/54) of the AEs were reported as serious AEs (SAEs), all of them reported as unrelated or unlikely to be related to lucinactant administration. All SAEs are detailed in Table 3, including onset date and study day when the death occurred, if applicable. In total, 37% (7/19) of the patients in the study died. Most deaths were related to sepsis or infection, and all but 1 occurred after study day 13. None of the deaths were reported as related to the study intervention. Pneumothorax was reported in 11% (2/19) of the participants, which occurred more than 20 days after drug administration.

**Table 2.** Study outcome data (N=20).

	Baseline	12 hours	24 hours	Day 5
Physiological parameters				
OI^a^, mean (SD)	10.4 (5.8)	8.6 (2.7)	8.5 (4.1)	5.7 (2.4)
P/F^b^, mean (SD)	193 (68.6)	179 (57.4)	192 (61.4)	223 (105.0)
S/F^c^, mean (SD)	182 (55.8)	199 (40.6)	200 (52.2)	210 (65.7)
C_rs_^d^, mean (SD)	39.3 (16.6)	34.1 (8.2)	40.5 (11.1)	52.9 (17.5)
Clinical outcome measures				
Days on MV^e^, mean (SD)	14.3 (10.2)	—^f^	—	—
MV-free days, mean (SD)	10.3 (12.1)	—	—	—
Days in the ICU^g^, mean (SD)	18.6 (9.4)	—	—	—
Days in the hospital, mean (SD)	21.9 (8.3)	—	—	—
Progressed to ECMO^h^, n (%)	2 (10)	—	—	—

a OI: oxygenation index.

b P/F: PO_2_-to–fraction of inspired oxygen (FiO_2_) ratio.

c S/F: oxygen saturation–to-FiO_2_ ratio.

d C_rs_: respiratory system compliance.

e MV: mechanical ventilation.

f Not available.

g ICU: intensive care unit.

h ECMO: extracorporeal membrane oxygenation.

**Table 3.** Serious adverse events.

Patient and serious adverse event (term reported)	Onset (study day)	Death (study day)
Patient A (aged 69 years)		24
Acute kidney injury	9	
Atrial fibrillation	6	
Bacterial staphylococcal infection	22	
Fungal blood infection	3	
MSSA^a^ pneumonia	8	
Pneumothorax	21	
Pulmonary embolus	15	
Worsening COVID-19 or pneumonia	15	
Patient B (aged 72 years)		22
Septic shock	11	
Multiorgan failure	11	
Patient C (aged 25 years)		
ECMO^b^ deployment	1	—^c^
Patient D (aged 59 years)		
Bacteremia or fungemia	8	14
Patient E (aged 51 years)		
Sepsis due to COVID-19 pneumonia	6	13
Patient F (aged 56 years)		
Cardiac arrest	3	3
Patient G (aged 73 years)		—
Bradycardia	N/A^d^	
Pneumonia	N/A	
Septic shock	N/A	
Severe hyperkalemia	N/A	
Patient H (aged 62 years)		19
Septic shock to respiratory focus	18	
Ventilator pneumonia	6	
Patient I (aged 50 years)		
Streptococcal pneumonia	5	25
Patient J (aged 67 years)		—
Pneumothorax	N/A	
Septic shock	N/A	

a MSSA: methicillin-sensitive *Staphylococcus aureus*.

b ECMO: extracorporeal membrane oxygenation.

c Not applicable.

d N/A: not available.

## Discussion

### Principal Findings

In this study, we tested the effects of a different surfactant preparation on acute lung injury caused by COVID-19–associated ARDS. Patients with COVID-19–associated ARDS have primary surfactant deficiency and dysfunction that lead to severe atelectasis and intrapulmonary shunting [8,27]. SARS-CoV-2 possesses a spike protein that targets alveolar type 2 cells and decreases the synthesis and secretion of surfactant. The secreted surfactant is further inactivated by influxing plasma proteins and immunoglobulin A autoantibodies against pulmonary surfactant proteins B and C in some patients with severe COVID-19 [11]. The disruption of surfactant production and the function of secreted surfactant resulted in an increased number of patients with very severe hypoxemia and very noncompliant lungs compared to non–COVID-19 ARDS. Restoration of surfactant presence could potentially interrupt this vicious cycle. Prior clinical trials and case reports of surfactant administration to adults with non–COVID-19 ARDS improved short-term oxygenation [13-15,28,29] but did not affect longer-term outcomes such as time to extubation or survival [16]. While the reasons for the limited effects are multiple [30], one possibility is the inability to overcome the dysfunction of secreted surfactant through an exogenous surfactant [31]. Because primary surfactant deficiency is a major pathophysiologic mechanism in COVID-19–associated ARDS, there is greater likelihood that an exogenous surfactant can decrease surface tension in the lungs of these patients [32,33].

Lucinactant, a peptide-containing synthetic surfactant that has been approved by the US Food and Drug Administration to treat neonatal RDS, has been shown in a series of in vitro studies to be more resistant than animal-derived surfactants to the inhibitory effects of plasma proteins, including fibrinogen [34,35], as well as to inactivation by hypochlorous acid and reactive oxygen species [36]. In addition, lucinactant has been shown to modulate the inflammatory response in various animal models of lung injury, including murine H1N1 influenza, hyperoxia, and lipopolysaccharide lung injury in mice [37], and acute lung injury in pigs [38].

Lucinactant has been studied in phase 1 and phase 2 clinical trials in adult patients with non–COVID-19 ARDS [13]. In these studies, lucinactant was delivered to multiple lung segments via a bronchoscope, resulting in short-term improvement in oxygenation. In this study, we chose to administer lucinactant as liquid boluses via intratracheal instillation to right and left bronchi through an ETT to minimize peridosing desaturation in these patients with severe mechanical and gas exchange abnormalities. Intratracheal instillation also simplified the treatment setup for patients who were under strict respiratory and contact isolations and decreased exposure risk to COVID-19 for the clinical and research staff compared to bronchoscopic instillation. Aerosolization would have been a preferred method, but an optimal aerosolizer to deliver lucinactant was not available for adult patients at the time of the study. By using intratracheal instillation, we achieved a good tolerance of the volumes of the drug delivered to the lung units. Dosing was interrupted or discontinued due to peridosing events only on 12.5% (3/24) of the dosing procedures. The occurrence of peridosing events using this administration approach was low and mostly transient and mild.

Most of the reported AEs and SAEs were expected for this population with severe illness, and most were not related or unlikely to be related to the study therapy. The occurrence of air leaks has been reported in previous surfactant non–COVID-19 ARDS trials. In our study, there were 2 events of pneumothorax that required the placement of a chest tube. Both occurred after study day 20; thus, they were unlikely to be related to lucinactant dosing. There were 7 deaths in this study. Six occurred >13 days after lucinactant administration, whereas 1 patient died of cardiac arrest on study day 3. The mortality rate in this study (7/19, 36.8%) was within the expected range for patients with severe ARDS during the early to mid–COVID-19 pandemic [39].

The primary efficacy end point, OI from baseline to 5 days after dosing, showed no improvement. Other gas exchange parameters, such as PO_2_-to-FiO_2_ and oxygen saturation–to-FiO_2_ ratios, PO_2_, and PaCO_2_, also did not change. Respiratory system compliance (C_rs_) tended to increase from baseline to study day 5 by approximately 35%, but the change did not reach statistical significance. Because the patients were all heavily sedated, the improvement in respiratory system compliance implied that the impaired lung compliance of ARDS may have been attenuated by lucinactant treatment. The reasons for the lack of significant oxygenation and lung mechanics improvement in this study may be multiple, including insufficient dosing (only 5/19, 26.3% of the patients received repeated doses) and the relatively late dosing (within 7 days after MV initiation). The logistics for delivering multiple doses were made difficult by the stringent isolation protocols imposed on patients with COVID-19 and their caretakers in each institution. Earlier dosing, larger doses, or repeated surfactant doses (eg, via continuous aerosolization) could increase the amount of lucinactant delivered to atelectatic alveoli, and therefore, yield more significant improvements in gas exchange and lung mechanics.

Some case reports of surfactant treatment for COVID-19–related ARDS have been recently published, showing encouraging results [28,33,40,41], and at least 4 randomized studies were initiated during the pandemic [20,42-44]. To our knowledge, only 1 of them has been published [20]. This study included 12 patients treated with nebulized surfactant and 8 controls and showed no difference in oxygenation between the groups at 48 hours [20]. One study has been terminated due to a low recruitment rate, and 2 have no results posted on ClinicalTrials.gov.

Most of the patients in our study (17/20, 85%) received corticosteroids, mostly before lucinactant administration and, in some cases (5/20, 25%), before and after lucinactant instillation. The use of steroids is a common practice in patients with severe COVID-19. Steroids have been shown to reduce inflammation and decrease the immune-mediated lung injury, resulting in decreased mortality in these patients [45]. Remdesivir was used in 25% (5/20) of the patients. Extracorporeal membrane oxygenation was deployed in 10% (2/20) of the patients, with 1 dying of worsening COVID-19 and septic shock. As our trial did not standardize the use of these medications, we cannot draw any conclusions on how these treatments may have impacted our results. Other limitations of this study include the small sample size and the lack of a control group. Similarly, lack of blinding may have influenced ventilatory management and clinical care; however, we did achieve the key objective of this pilot study, which was to demonstrate the feasibility and safety of delivering lucinactant via intratracheal instillation to patients with severe COVID-19–associated ARDS who were in strict isolation. This provided valuable information on the design of future studies involving patients with ARDS with severe surfactant deficiency [46].

There are several other explanations for why the results were not significant. Steroids, remdesivir, and extracorporeal membrane oxygenation were used in many patients. This could decrease the positive effects of surfactant such that a much larger patient population would be required to demonstrate them. The technique used to deliver surfactant in our study might not deliver enough to the distal lung regions. Delivering the surfactant using a bronchoscope could be more effective, but bronchoscopy was considered a procedure with the highest risk of COVID-19 transmission and, therefore, was difficult to arrange. Surfactant could also be delivered more effectively into the lung through aerosolization, but the device was not available at the time of the study.

Finally, this study also highlighted the challenges of executing a pulmonary intervention in a raging pandemic during which significant barriers must be placed between the patients and caregivers. These circumstances may limit the recruitment and the delivery of therapies that require repeated dosing, such as in our study. A more convenient delivery method that decreases the need to break the barriers, such as continuous aerosolization, would facilitate the execution of these types of clinical studies.

### Conclusions

In this small multicenter, single-arm, open-label study, we showed that 1 to 2 doses of lucinactant administered via intratracheal instillation in severe ARDS due to COVID-19 were generally well tolerated. The incidence of postdosing events was low in comparison to other surfactant preparations in previous ARDS trials. There were no statistically significant changes in respiratory system compliance and parameters of oxygenation during the first 5 days. Our data suggest that, when future studies of lucinactant are conducted in patients with ARDS with severe surfactant deficiency, an earlier and longer treatment protocol using different delivery methods that reach a larger alveolar surface area, such as aerosolization, may be needed to yield any beneficiary effects.

## Supplementary material

10.2196/77315 Checklist 1 CONSORT checklist.

## Abbreviations

AE: adverse event
ARDS: acute respiratory distress syndrome
CONSORT: Consolidated Standards of Reporting Trials
ETT: endotracheal tube
FiO_2_: fraction of inspired oxygen
ICU: intensive care unit
MV: mechanical ventilation
OI: oxygenation index
PaCO_2_: partial pressure of carbon dioxide in arterial blood
RDS: respiratory distress syndrome
SAE: serious adverse event
SpO_2_: peripheral oxygen saturation

## References

[1] WHO COVID-19 dashboard. World Health Organization.

[2] Guan WJ, Ni ZY, Hu Y (2020). Clinical characteristics of coronavirus disease 2019 in China. N Engl J Med.

[3] Oud L, Garza J (2023). The contribution of COVID-19 to acute respiratory distress syndrome-related mortality in the United States. J Clin Med Res.

[4] Kollef MH, Schuster DP (1995). The acute respiratory distress syndrome. N Engl J Med.

[5] Bernard GR, Artigas A, Brigham KL (1994). The American-European Consensus Conference on ARDS. Definitions, mechanisms, relevant outcomes, and clinical trial coordination. Am J Respir Crit Care Med.

[6] Gattinoni L, Busana M, Camporota L, Marini JJ, Chiumello D (2021). COVID-19 and ARDS: the baby lung size matters. Intensive Care Med.

[7] Gattinoni L, Meissner K, Marini JJ (2020). The baby lung and the COVID-19 era. Intensive Care Med.

[8] Schousboe P, Ronit A, Nielsen HB (2022). Reduced levels of pulmonary surfactant in COVID-19 ARDS. Sci Rep.

[9] Camporota L, Chiumello D, Busana M, Gattinoni L, Marini JJ (2021). Pathophysiology of COVID-19-associated acute respiratory distress syndrome. Lancet Respir Med.

[10] Calkovska A, Kolomaznik M, Calkovsky V (2021). Alveolar type II cells and pulmonary surfactant in COVID-19 era. Physiol Res.

[11] Sinnberg T, Lichtensteiger C, Ali OH (2023). Pulmonary surfactant proteins are inhibited by immunoglobulin a autoantibodies in severe COVID-19. Am J Respir Crit Care Med.

[12] Spragg RG, Gilliard N, Richman P (1994). Acute effects of a single dose of porcine surfactant on patients with the adult respiratory distress syndrome. Chest.

[13] Wiswell TE, Smith RM, Katz LB (1999). Bronchopulmonary segmental lavage with Surfaxin (KL(4)-surfactant) for acute respiratory distress syndrome. Am J Respir Crit Care Med.

[14] Spragg RG, Lewis JF, Walmrath HD (2004). Effect of recombinant surfactant protein C-based surfactant on the acute respiratory distress syndrome. N Engl J Med.

[15] Spragg RG, Taut FJ, Lewis JF (2011). Recombinant surfactant protein C-based surfactant for patients with severe direct lung injury. Am J Respir Crit Care Med.

[16] Willson DF, Truwit JD, Conaway MR, Traul CS, Egan EE (2015). The adult calfactant in acute respiratory distress syndrome trial. Chest.

[17] Davidson WJ, Dorscheid D, Spragg R, Schulzer M, Mak E, Ayas NT (2006). Exogenous pulmonary surfactant for the treatment of adult patients with acute respiratory distress syndrome: results of a meta-analysis. Crit Care.

[18] Bhatt RM, Clark HW, Girardis M, Busani S (2021). Exogenous pulmonary surfactant in COVID-19 ARDS. The similarities to neonatal RDS suggest a new scenario for an “old” strategy. BMJ Open Respir Res.

[19] Khudadah K, Ramadan A, Othman A (2023). Surfactant replacement therapy as promising treatment for COVID-19: an updated narrative review. Biosci Rep.

[20] Dushianthan A, Clark HW, Brealey D (2023). A randomized controlled trial of nebulized surfactant for the treatment of severe COVID-19 in adults (COVSurf trial). Sci Rep.

[21] Cochrane CG, Revak SD (1991). Pulmonary surfactant protein B (SP-B): structure-function relationships. Science.

[22] (2005). Lucinactant in neonatal respiratory distress syndrome: profile report. Drugs Ther Perspect.

[23] Moya FR, Gadzinowski J, Bancalari E (2005). A multicenter, randomized, masked, comparison trial of lucinactant, colfosceril palmitate, and beractant for the prevention of respiratory distress syndrome among very preterm infants. Pediatrics.

[24] Sinha SK, Lacaze-Masmonteil T, Valls i Soler A (2005). A multicenter, randomized, controlled trial of lucinactant versus poractant alfa among very premature infants at high risk for respiratory distress syndrome. Pediatrics.

[25] Laughon MM, Smith PB, Bose C (2009). Prevention of bronchopulmonary dysplasia. Semin Fetal Neonatal Med.

[26] Thomas NJ, Guardia CG, Moya FR (2012). A pilot, randomized, controlled clinical trial of lucinactant, a peptide-containing synthetic surfactant, in infants with acute hypoxemic respiratory failure. Pediatr Crit Care Med.

[27] Richmond BW, Dela Cruz CS (2023). Adding insult to injury: does COVID-19 promote acute respiratory distress syndrome by inhibiting surfactant?. Am J Respir Crit Care Med.

[28] Heikinheimo M, Hynynen M, Rautiainen P, Andersson S, Hallman M, Kukkonen S (1994). Successful treatment of ARDS with two doses of synthetic surfactant. Chest.

[29] Imperatore F, Postiglione M, Diurno F, Cirillo F, Liguori G, Pica M (1999). The instillated surfactant in the treatment of sepsis-induced ARDS: case report. Intensive Care Med.

[30] Gadek JE (1996). Consensus on surfactant and inhaled nitric oxide for ARDS. J Aerosol Med.

[31] Markart P, Ruppert C, Wygrecka M (2007). Patients with ARDS show improvement but not normalisation of alveolar surface activity with surfactant treatment: putative role of neutral lipids. Thorax.

[32] Cattel F, Giordano S, Bertiond C (2021). Use of exogenous pulmonary surfactant in acute respiratory distress syndrome (ARDS): role in SARS-CoV-2-related lung injury. Respir Physiol Neurobiol.

[33] Heching M, Lev S, Shitenberg D, Dicker D, Kramer MR (2021). Surfactant for the treatment of ARDS in a patient with COVID-19. Chest.

[34] Manalo E, Merritt TA, Kheiter A, Amirkhanian J, Cochrane C (1996). Comparative effects of some serum components and proteolytic products of fibrinogen on surface tension-lowering abilities of beractant and a synthetic peptide containing surfactant KL4. Pediatr Res.

[35] Amirkhanian JD, Merritt TA (1998). Inhibitory effects of oxyradicals on surfactant function: utilizing in vitro Fenton reaction. Lung.

[36] Merritt TA, Amirkhanian JD, Helbock H, Halliwell B, Cross CE (1993). Reduction of the surface-tension-lowering ability of surfactant after exposure to hypochlorous acid. Biochem J.

[37] Kinniry P, Pick J, Stephens S (2006). KL4-surfactant prevents hyperoxic and LPS-induced lung injury in mice. Pediatr Pulmonol.

[38] Zimmermann AM, Roberts KD, Lampland AL (2010). Improved gas exchange and survival after KL-4 surfactant in newborn pigs with severe acute lung injury. Pediatr Pulmonol.

[39] Meyer NJ, Gattinoni L, Calfee CS (2021). Acute respiratory distress syndrome. Lancet.

[40] Busani S, Dall’Ara L, Tonelli R (2020). Surfactant replacement might help recovery of low-compliance lung in severe COVID-19 pneumonia. Ther Adv Respir Dis.

[41] Piva S, DiBlasi RM, Slee AE (2021). Surfactant therapy for COVID-19 related ARDS: a retrospective case-control pilot study. Respir Res.

[42] (2021). Randomized controlled phase II trial of poractant alfa (Curosurf®) by fiberoptic bronchoscopy-directed endobronchial administration in acute respiratory distress syndrome (ARDS) due to COVID-19 viral pneumonia. National Institutes of Health.

[43] (2021). Phase I/II trial: exogenous surfactant administration for patients with COVID-19. National Institutes of Health.

[44] (2023). Multicenter, open-label, randomised trial to assess the efficacy and tolerability of poractant alfa(porcine surfactant, Curosurf®) in hospitalized patients with SARS-COV-19 acute respiratory distress syndrome (ARDS). National Institutes of Health.

[45] Chaudhuri D, Sasaki K, Karkar A (2021). Corticosteroids in COVID-19 and non-COVID-19 ARDS: a systematic review and meta-analysis. Intensive Care Med.

[46] Kula R, Maca J, Sklienka P (2011). Exogenous surfactant as a component of complex non-ECMO therapy of ARDS caused by influenza A virus (2009 H1N1). Bratisl Lek Listy.

